# Beta-cell replacement for type 1 diabetes: integrating cell products, engraftment and immune protection

**DOI:** 10.3389/fendo.2026.1981945

**Published:** 2026-10-05

**Authors:** Lan Chen, Xinhao Hu, Chen Zheng, Qi Huang, Jiani Xu, Xiongbin You, Chenfang Wang

**Affiliations:** 1 The Third Hospital Affiliated to Zhejiang Chinese Medical University, Hangzhou, China; 2 Zhejiang Chinese Medical University, Hangzhou, China; 3 The First Hospital Affiliated to Zhejiang Chinese Medical University, Hangzhou, China

**Keywords:** beta-cell replacement, functional cure, immune protection, stem-cell-derived islets, transplantation, type 1 diabetes

## Abstract

Beta-cell replacement aims to restore endogenous, glucose-responsive insulin secretion in type 1 diabetes. Donor-islet transplantation has shown that this approach can improve glycaemic control and prevent severe hypoglycaemia. Its wider use is limited by donor supply, early graft loss and chronic systemic immunosuppression. Pluripotent stem cells provide a renewable source of pancreatic progenitors and differentiated islet products. Recent clinical studies have reported C-peptide secretion and insulin independence in some recipients. However, cell supply is only one part of the problem. Product maturity, graft composition, implantation site, vascular support, immune injury and manufacturing quality all affect outcome. This review examines these factors as parts of one treatment system. It compares donor islets, stem-cell-derived products and islet-like organoids, then evaluates delivery and immune-protection strategies. It also considers clinical endpoints, patient selection and safety. We define functional cure as durable endogenous insulin secretion with physiological benefit at an acceptable total treatment burden. Current evidence provides early evidence of clinical activity and, in selected recipients, clinically meaningful efficacy. However, the available studies involve highly selected participants, small cohorts and/or systemic immunosuppression. No platform has yet combined durable function, immune control, scalable production and acceptable long-term safety.

## 1 Introduction

Type 1 diabetes (T1D) results from immune-mediated destruction of pancreatic beta cells (1, 2). The loss of endogenous insulin secretion disrupts physiological glucose control and makes lifelong insulin replacement necessary (3). Exogenous insulin can limit hyperglycaemia, but it cannot reproduce the rapid and local responses of native beta cells. People with T1D therefore remain at risk of severe hypoglycaemia, glycaemic variability and long-term vascular complications. The central therapeutic problem is the loss of a functional beta-cell population.

Insulin analogues, continuous glucose monitoring and automated insulin-delivery systems have improved daily management and reduced treatment risk (4). However, these approaches compensate for insulin deficiency rather than restoring beta-cell function or removing the autoimmune process. They also require continuous monitoring, device use or dose adjustment. Better disease management should therefore not be equated with cure.

Beta-cell replacement has a different goal: to restore a living endocrine graft that senses glucose and releases insulin when needed (5). Donor-islet transplantation provides clinical proof of this principle. In selected people with T1D, it can improve glycaemic control, prevent severe hypoglycaemia and sometimes remove the need for exogenous insulin (6). Its broader use is limited by donor scarcity, variable graft quality, early cell loss and chronic systemic immunosuppression (6–8). Pluripotent stem-cell (PSC)-derived products may provide a renewable and more standardised source of beta cells (9–11), but cell supply alone is not enough. A useful graft must mature, engraft, become vascularised and resist both alloimmune rejection and recurrent autoimmunity (12–14). Cell replacement and immune protection must therefore be developed as parts of the same therapy.

This review examines donor islets and pluripotent stem-cell-derived beta-cell products. It then considers the conditions that determine whether a durable graft can be achieved: product maturation, manufacturing, delivery, vascularisation and immune protection. It also evaluates clinical evidence, outcome measures, patient selection, safety and regulatory readiness. Throughout, success is judged by durability, physiological benefit and total treatment burden. Short-term insulin independence or graft function maintained only by indefinite systemic immunosuppression remains an important advance, but not a broadly applicable cure.

Throughout this review, a beta-cell product denotes a preparation designed primarily to restore beta-cell function. An islet-like or multicellular product contains beta cells with additional endocrine cell types and may recapitulate selected islet interactions. We use pluripotent stem-cell (PSC)-derived as an umbrella term for products derived from human embryonic stem cells (hESCs) or induced pluripotent stem cells (iPSCs). These labels distinguish product composition from the specific clinical function being evaluated. Recent reviews have focused on engineering stem-cell products and their translational barriers (15), or on immunological barriers and graft-directed immune modulation (16, 17). Here, we use a complementary comparison framework that evaluates the cell product, the early engraftment window and implant niche, immune-protection strategy, manufacturing and long-term safety, clinical endpoints, and treatment burden as interdependent parts of one therapeutic system (**Figure 1**).

**Figure 1:**
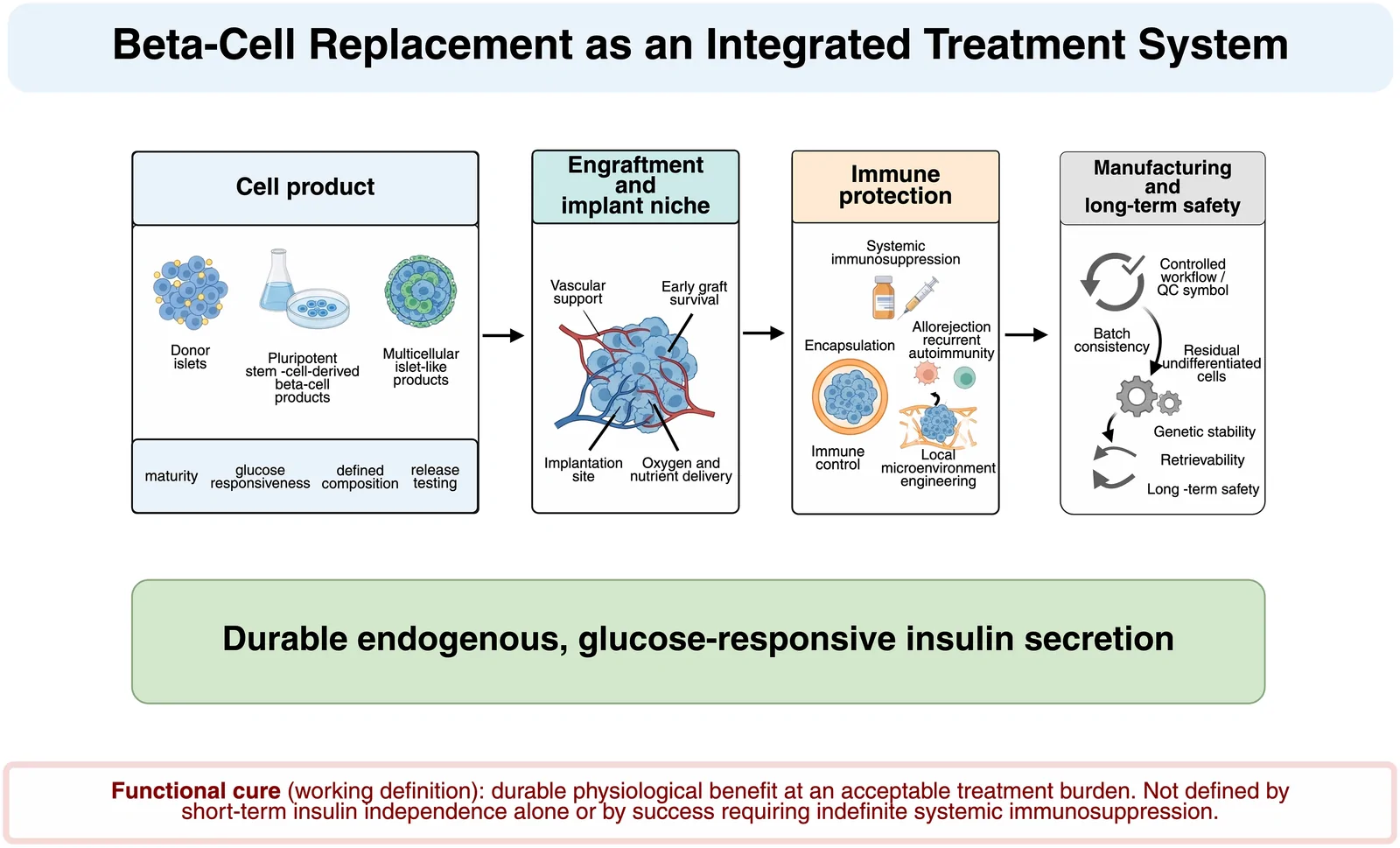
Beta-cell replacement should be evaluated as an integrated treatment system. Durable clinical benefit depends on a functional cell product, successful engraftment, effective immune protection, and scalable manufacturing with long-term safety control. The working definition of functional cure requires sustained endogenous insulin secretion with physiological benefit at an acceptable treatment burden.

## 2 Review scope and literature selection

This narrative review integrates evidence on donor-islet and PSC-derived beta-cell replacement in T1D, with emphasis on engraftment, immune protection and clinical translation. The synthesis prioritises original mechanistic and transplantation studies, human clinical reports and long-term cohorts; reviews provide conceptual context. Studies of other disease settings are considered only when they inform cell manufacture or a mechanism directly relevant to beta-cell replacement. Reports unrelated to these questions are outside the scope. This is not a systematic review or meta-analysis, and no pooled effect estimates are calculated.

We searched PubMed, Web of Science Core Collection, and Embase from database inception to September 1, 2026. The database searches combined terms for type 1 diabetes and pancreatic islets or beta cells with terms related to cell transplantation, stem-cell-derived products, engraftment, graft survival, vascularisation, and immune protection. Controlled vocabulary terms and free-text terms were used in combination. The complete search strategies for each database are provided in Supplementary 1. Because a single database may not capture all relevant studies, we also examined the reference lists of key original studies and relevant reviews and performed targeted searches for specific cell products, clinical development programs, and trial registration numbers.

The database searches identified 3,396 records, including 1,531 from PubMed, 872 from Web of Science Core Collection, and 993 from Embase. After 171 duplicate records were removed, 3,225 unique records remained. As this article is a narrative review, the search was intended to broaden the coverage of relevant evidence and support topic-based identification of the literature rather than to formally screen and include all retrieved records according to systematic review methodology. Accordingly, no formal risk-of-bias assessment or quantitative meta-analysis was performed.

During subsequent evidence selection and citation, priority was given to *in vitro* studies, animal studies, non-human primate studies, and human studies that directly addressed β-cell or islet replacement therapy for type 1 diabetes. Review articles were mainly used to provide background information, summarise developments in the field, and identify relevant original studies. Studies focused solely on general diabetes management, exogenous insulin therapy, glucose monitoring, or disease risk prediction, without directly addressing β-cell or islet replacement therapy, were not considered core evidence for this review.

## 3 Beta-cell replacement is necessary in type 1 diabetes

### 3.1 Beta-cell deficiency is the central pathological defect in T1D

Progressive loss of functional beta-cell mass is the defining defect in T1D. As beta cells are the body’s primary physiological source of insulin, no exogenous system can fully replicate their response to changing glucose (1, 18–20). Even when intensive treatment achieves acceptable glycaemic control, it cannot restore intrinsic glucose sensing or normal regulation of insulin output. Replacing lost beta-cell mass is therefore a biological requirement for restoring endogenous endocrine function, not simply a way of lowering glucose.

### 3.2 Immune intervention alone cannot fully restore endocrine function

Immune intervention can alter disease progression in some settings, especially when residual beta-cell function is still present (21). However, it cannot rebuild the functional beta-cell mass already lost in established T1D. This boundary matters: immunotherapy may slow further injury, but it does not by itself restore physiological insulin secretion. A durable restorative treatment will therefore need both immune control and a source of functional insulin-producing cells.

### 3.3 Donor islet transplantation provides proof of principle

Donor-islet transplantation provides the clearest clinical proof that beta-cell replacement can restore endogenous function. In selected people with recurrent severe hypoglycaemia, it reduces severe events, improves glycaemic stability and may achieve insulin independence (6, 7, 22, 23). At the same time, donor scarcity, early graft loss, variable long-term function and systemic immunosuppression prevent broad use. These constraints define the task for newer products: they must provide comparable metabolic benefit without relying on scarce donor tissue or imposing an unacceptable lifelong treatment burden.

## 4 Cellular sources for beta-cell replacement

### 4.1 Donor islets remain the clinical benchmark

Donor islets remain the reference product for beta-cell replacement. They contain mature endocrine cells arranged in a native islet-like structure and can release insulin in response to glucose soon after engraftment. Their clinical performance defines the metabolic benefit that newer products should aim to match (6).

Their value extends beyond insulin secretion. Donor islets provide a practical benchmark for product maturity, graft composition and early endocrine recovery. A stem-cell-derived product does not need to copy every feature of a donor islet. It should, however, show comparable glucose responsiveness and stable endocrine identity before claims of functional equivalence are made.

The limitations of donor tissue are equally informative. Suitable pancreata are scarce, and more than one donor may be required to achieve an adequate islet dose. Islet yield and quality vary with donor characteristics, procurement and ischaemic injury. Early loss after transplantation further reduces the functional graft mass. Donor islets are therefore an essential clinical benchmark, but not a scalable solution for most people with T1D (6–8).

### 4.2 Pluripotent stem-cell-derived beta cells provide a scalable alternative

Human embryonic stem cells and induced pluripotent stem cells can be expanded and directed through definitive endoderm, pancreatic progenitor and endocrine stages (12, 24). This developmental logic offers a renewable source of beta-cell-like products and could support more standardised manufacturing than donor-derived islets (25). It also allows the final product to be designed as progenitors, more mature endocrine cells or a defined multicellular islet-like cluster.

Preclinical studies have shown that stem-cell-derived pancreatic progenitors and more mature beta-like cells can engraft, release human C-peptide and improve glucose control (9–11). Early clinical studies have now extended this concept to people with T1D. Stem-cell-derived pancreatic products have shown graft survival and glucose-responsive C-peptide secretion after transplantation (26).

A renewable starting material does not automatically create a uniform medicine. The final product must retain function after storage, delivery and engraftment, while meeting defined limits for off-target cells and residual proliferative cells (12, 14, 27). Scalable supply is therefore only one part of the advantage of pluripotent stem cells. Product maturity, release testing and long-term safety will determine their clinical value.

### 4.3 ESC-derived and iPSC-derived products each have distinct advantages and limitations

hESC-derived products are well suited to large-scale, banked manufacture. Their differentiation pathways are extensively studied, and a single starting line can support a standardised allogeneic product (28). This model could simplify quality control and reduce the time between manufacture and treatment. However, allogeneic grafts remain exposed to rejection, while incomplete differentiation and residual proliferative cells require stringent release testing (14, 27).

iPSC-derived products create different opportunities. They provide a route to autologous treatment and avoid embryo-related concerns (29, 30). A first-in-human autologous iPSC-derived islet study reported endogenous insulin secretion and insulin independence in one participant (31). The result supports feasibility, but does not establish a routine personalised manufacturing pathway.

Autologous cells may reduce classical allorecognition, but they remain vulnerable to the autoimmune process that caused T1D. Patient-specific manufacture also requires reprogramming, expansion, differentiation and quality control for each recipient. Reprogramming-associated changes, mixed endocrine identity and the safety of immune-evasion edits must be assessed before clinical use (14, 27, 32). The relevant choice is therefore not simply autologous versus allogeneic. It is which manufacturing and immune strategy best fits the intended patient population.

### 4.4 Functional maturation and native-like architecture remain critical hurdles

Insulin expression alone does not define a transplantable beta-cell product. Cells must sense glucose across a physiological range, process proinsulin efficiently and release insulin with appropriate timing. They must also retain endocrine identity after implantation (12, 33). These features determine whether a graft can reduce glucose variability rather than merely add a small amount of C-peptide.

Three-dimensional organisation may help achieve this state. Islet-like organoid products and reaggregated stem-cell-derived islets aim to preserve cell-cell contacts that are difficult to reproduce in dispersed cultures. In preclinical cell-culture and transplantation studies, endocrine-cell clustering, prolonged maturation and improved alpha-beta cell contacts have been associated with better glucose-dependent insulin secretion (12, 33, 34). These studies support organoid-like organisation as a product-design strategy, not as evidence that every three-dimensional product is superior.

A more complex product also creates new constraints. Aggregate size can limit oxygen delivery, mixed cell composition can complicate release testing, and the product must remain consistent across batches. Its benefit should therefore be demonstrated by improved graft function, survival and reproducibility after transplantation. Native-like architecture is valuable only if it improves the complete therapeutic product rather than its appearance in culture.

## 5 Immune barriers to durable engraftment

### 5.1 Alloimmune rejection limits survival of transplanted cells

Donor islets and allogeneic stem-cell-derived products are recognised as foreign tissue. Host T cells, antibodies, natural killer cells and innate immune cells can each contribute to graft injury (14, 32, 35–38). The relative importance of these pathways depends on the cell product, transplant site and immunosuppressive regimen. Nevertheless, a graft that performs well in an immune-deficient model may fail rapidly in an immunocompetent recipient.

Clinical transplantation supports the relevance of donor-recipient immune compatibility. In a retrospective cohort of 48 islet-transplant recipients, at least one HLA-B match was associated with longer allograft survival (35). HLA matching alone is not sufficient, but the finding shows that allorecognition remains relevant even under systemic immunosuppression. A durable protection strategy must therefore control several immune pathways rather than a single T-cell pathway.

### 5.2 Autoimmune recurrence is a T1D-specific obstacle

T1D adds a second immune problem: the autoreactive immune memory that destroyed the recipient’s endogenous β cells can recur against a replacement graft. This risk may persist even when the graft is autologous or closely HLA matched, because HLA matching primarily reduces alloimmune recognition rather than β-cell-directed autoimmunity (39). Replacing cells therefore does not remove the disease process that caused their loss.

Alloimmunity and autoimmunity should not be treated as interchangeable. In human islet transplantation, monitoring of both alloantibodies and islet autoantibodies predicted clinical outcome (36). Autoantibodies are mainly markers of immune activity rather than the sole cause of beta-cell destruction. Their recurrence nevertheless shows why control of allorejection alone is unlikely to provide lasting function in T1D.

### 5.3 Early graft loss is driven by inflammation, hypoxia and poor vascularisation

Immune injury begins before classical rejection is established. Implantation exposes cells to hypoxia, nutrient deprivation, oxidative stress and inflammatory cytokines while local vascular connections are still forming. In intraportal islet transplantation, an instant blood-mediated inflammatory reaction can destroy cells soon after infusion. This early response is relevant even for autologous or immune-matched products because it does not require an HLA mismatch (40).

The liver remains the clinical reference site for islet transplantation because intraportal infusion is established and can provide benefit in selected people with T1D (6). However, islets enter the portal circulation in direct contact with blood rather than a prepared tissue bed. Coagulation and inflammatory pathways are activated at this stage, and the remaining cells must then survive a period of limited oxygen supply before vascular integration. Primary graft function at 28 days was associated with outcomes at 5 years in a multicentre registry analysis, underlining the clinical importance of this early window (7).

These losses reduce the functional graft mass available for long-term engraftment. They also make later immune-control strategies harder to interpret, because poor function may reflect inadequate early survival rather than failure of a specific immune intervention. Improving the implantation microenvironment is therefore part of immune protection, not a separate technical detail.

Extrahepatic sites are being explored for this reason. The anterior rectus sheath supported maturation and function of human pluripotent stem-cell-derived islets in non-human primates (13). The same site was later used for autologous chemically induced iPSC-derived islets in a first-in-human study (31). Subcutaneous, intramuscular and omental sites provide different balances of surgical access, tissue volume, vascular supply and local immune exposure. An accessible site may also permit graft monitoring or removal, which is important for an encapsulated or gene-edited product. Yet accessibility does not guarantee prompt vascularisation; a scaffold, device or prevascularisation step may still be needed. Evidence for each site must therefore be interpreted within its species, cell product and immune regimen; the early human result does not establish superiority over other implantation sites.

The implantation site therefore constrains the protection method that can realistically work. A device with a good immune barrier but poor oxygen transport will not preserve a large graft. Conversely, rapid vascular access may improve cell survival while increasing immune exposure. Preclinical studies should report early cell survival, vascularisation and local inflammation alongside later C-peptide and glycaemic endpoints (**Figure 2**). This links the choice of site directly to the interpretation of immune-protection data.

**Figure 2:**
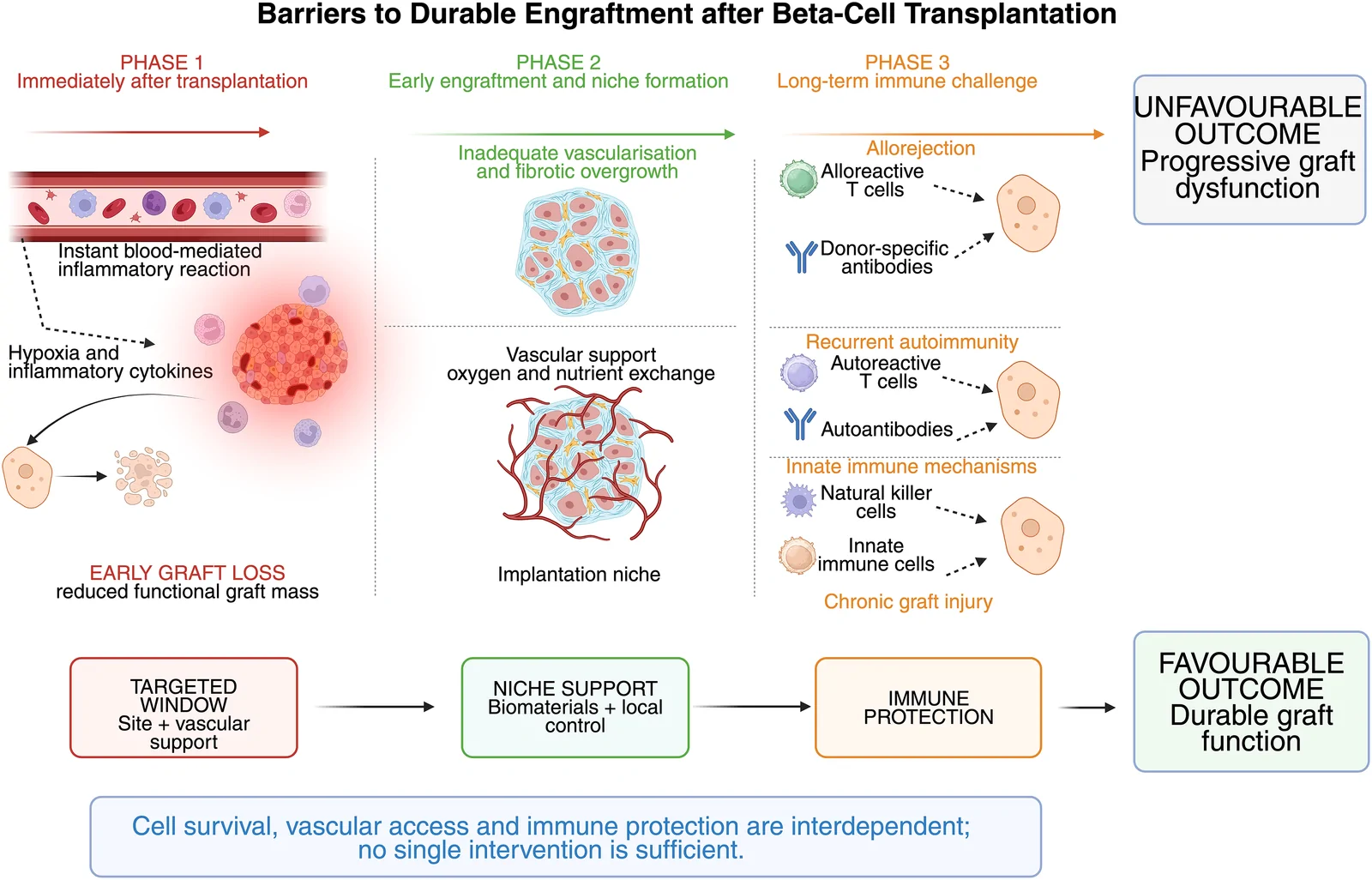
Time-dependent barriers to durable engraftment after beta-cell transplantation. Early inflammatory injury, implantation-site constraints and later immune attack act sequentially but overlap biologically. Durable function is likely to require interventions that address each phase as part of one treatment system.

### 5.4 Systemic immunosuppression is effective but not ideal

Systemic immunosuppression is the established way to sustain an allogeneic beta-cell graft. It has enabled donor-islet transplantation and remains the current clinical benchmark (5, 6). Its benefit, however, is obtained by adding a second long-term treatment that requires drug monitoring and is associated with risks of renal, metabolic, haematological and infectious complications (41–43).

These risks are not background concerns. In a donor-islet phase 3 trial, serious events related to the procedure or immunosuppression included cytopenias, infections and renal dysfunction (6). Neutropenia was also the most common serious adverse event in a recent stem-cell-derived islet study (5). Chronic systemic immunosuppression may remain reasonable for selected high-risk patients, but it is not an acceptable universal route to a functional cure.

This distinction sets the standard for evaluating newer approaches. A report of insulin independence is clinically meaningful, but it should be interpreted together with graft durability, endogenous insulin secretion, procedure-related risk and the burden of immune treatment. The next chapter considers strategies that aim to protect the graft more selectively.

## 6 Strategies for immune protection

No single strategy is likely to solve every form of graft injury. A useful approach must preserve endocrine function, limit innate and adaptive immune attack, and avoid imposing a treatment burden that defeats the purpose of cell replacement. The central question is therefore not which strategy is most sophisticated, but which combination can meet these conditions in a defined clinical setting.

Immune tolerance and immune evasion describe different objectives. Tolerance modifies the recipient response to permit graft acceptance, whereas evasion modifies the graft to reduce immune recognition or clearance. These approaches may be complementary: reduced graft recognition does not establish recipient tolerance, and tolerance-directed interventions may require continued control of inflammatory triggers. Recent reviews provide complementary accounts of these mechanisms and their integration with biomaterials (44, 45). **Table 1** compares their advantages, limitations, safety concerns and current evidence stages.

**Table 1:** Immune-protection and graft-support strategies for β-cell replacement in type 1 diabetes.

Strategy	Mechanism and principal advantage	Evidence stage and representative findings	Main limitations	Potential safety concerns	Role in combinations
Systemic immunosuppression	Broad control of alloreactivity; established clinical comparator.	Human phase 3 donor-islet evidence and phase 1/2 PSC-islet efficacy (5, 6).	Long-term drugs and monitoring; not antigen-specific.	Infection, cytopenias and renal/metabolic toxicity (6, 43).	May accompany local protection; selected high-risk recipients.
Encapsulation	Physical immune isolation; macrodevices can localise the graft.	Primate proof of concept (48); human device experience includes VX-264, not advanced after its efficacy endpoint was missed (49).	Oxygen/nutrient transport, fibrosis and device capacity; microcapsules are difficult to retrieve.	Foreign-body response, device failure and implantation/explantation complications (46).	May combine with local signals or vascular support.
Non-isolating biomaterials	Scaffolds or matrices retain cells and support the local niche without excluding immune cells.	Predominantly preclinical scaffold and islet-organoid studies (34).	Benefits depend on material, site and cell dose; no intrinsic control of allorejection.	Local inflammation and material-related tissue reactions require assessment.	Supports cells, devices or local immune modulation.
Local immunomodulation	Local delivery/presentation of signals may reduce systemic exposure.	CXCL12 capsules in mice (51); FasL microgels plus transient rapamycin in primates (52); CCL22 cell-based Treg recruitment in mice (53).	Dose, persistence and immune specificity; durable human efficacy unproven.	Unintended local immune suppression and delivery-system effects require testing.	May recruit endogenous Tregs; compatible with barrier or non-barrier systems.
Cell-intrinsic immune engineering	Reduces graft recognition and/or immune injury while aiming to preserve endocrine function.	Humanised-mouse and primate studies (37, 38); one human donor-islet case with 12-week function without systemic immunosuppression (57).	No established therapeutic-dose durability; residual immune pathways and function must be assessed.	Unintended genomic changes, abnormal growth and impaired immune surveillance (27, 58).	Can combine with local support and validated safety mechanisms.
Treg-based immune modulation	Recipient-directed suppression; antigen-specific or CAR-Tregs may focus activity.	Early human feasibility with polyclonal Tregs (21, 62); antigen-specific and CAR-Treg platforms remain preclinical (63, 64).	Persistence, localisation and functional stability; no proof of replacing chronic immunosuppression.	Unwanted immune suppression and instability of the regulatory phenotype require assessment (59, 60).	Adjunct to replacement grafts; distinct from endogenous recruitment.

### 6.1 Systemic immunosuppression remains the current clinical standard

Systemic immunosuppression remains the established clinical comparator for preventing allograft rejection (5, 6). In this review, it functions as the benchmark against which alternative protection strategies in **Table 1** should be judged. Its lack of antigen specificity and long-term burden limit broad use; it may remain appropriate for selected people with recurrent severe hypoglycaemia despite optimised care, or for recipients already receiving immunosuppression for another organ transplant (6, 8).

### 6.2 Encapsulation can physically separate grafts from the immune system

Encapsulation is a physical isolation strategy. This section considers materials only in their role as semipermeable barriers that restrict cellular immune access while permitting transport of glucose, nutrients and insulin. Their active effects on the implantation microenvironment are considered separately in Section 6.3.

The design choice is not simply the size of the device. Microcapsules provide a high surface-to-volume ratio and short diffusion distances, but they are difficult to retrieve individually. Macrodevices can localise, monitor and remove a defined graft, which is valuable for stem-cell-derived or gene-edited products. Their larger dimensions, however, can make oxygen delivery and uniform vascularisation more difficult (46, 47).

Transport is the central trade-off. A barrier that blocks immune cells can also slow oxygen and nutrient delivery, especially before vascularisation. Fibrotic overgrowth can further impair diffusion. In macaques, allogeneic islets within chemically modified alginate remained viable and glucose responsive for four months without systemic immunosuppression (48). This important result supports a specific material-site combination, not a general claim that encapsulation can maintain long-term glycaemic control in people with T1D.

Clinical macrodevices also differ in whether they provide immune isolation. PEC-01 pancreatic endoderm in VC-02/PEC-Direct uses a vascularising, non-immunoprotective device and requires systemic immunosuppression; it should not be confused with the immune-isolating VC-01/PEC-Encap platform (26). VX-264 tested fully differentiated islet cells within a proprietary immunoprotective device, aiming to avoid systemic immunosuppression. In its 28 March 2025 announcement, Vertex reported that the day-90 Part B analysis did not meet the efficacy endpoint and that further clinical development would not proceed (49). This sponsor-reported outcome illustrates the difficulty of translating a cells-plus-device strategy, but does not by itself establish the mechanism of inadequate function.

Device performance should therefore be assessed beyond cell survival. Relevant outcomes include dynamic C-peptide secretion, postprandial glucose control, fibrosis, device integrity, implantation and explantation burden, and the need for concurrent immune treatment. A living graft behind a barrier is not sufficient if glucose sensing or insulin delivery is too slow to provide meaningful metabolic benefit.

### 6.3 Non-isolating biomaterials can engineer the graft microenvironment

Here, biomaterial engineering refers to non-isolating approaches that improve the graft microenvironment. Hydrogels, scaffolds and engineered matrices can retain the graft, reduce local inflammation and support vascularisation (50). They may be combined with an encapsulation device, but their primary role is not physical immune exclusion.

Local immunomodulation can operate with or without a physical barrier. In immunocompetent diabetic mice, CXCL12-containing alginate microcapsules reduced immune-cell accumulation and supported human stem-cell-derived beta-cell function for more than 150 days (51). In diabetic non-human primates, Fas ligand-presenting microgels cotransplanted with allogeneic islets, together with a short course of rapamycin, supported C-peptide secretion and glycaemic control for more than six months (52). These studies suggest that local signals can reduce inflammatory and alloreactive pressure while preserving graft function.

The distinction from encapsulation is functional rather than material-based. Physical barriers are judged by immune isolation and mass transfer. Non-isolating materials are judged by their effects on vascularisation, inflammation and local delivery. The two approaches can be combined, but studies should report the contribution and limitation of each component separately.

Local signals can also recruit endogenous regulatory cells rather than supplying expanded Tregs. Oran et al. engineered human hepatic stellate cells to secrete CCL22 and combined them with MIN6 beta cells in heterospheroids; transplantation in mice increased local Treg recruitment (53). This preclinical cell-based delivery system links graft-microenvironment engineering to the Treg approaches in Section 6.5. Increased recruitment alone does not demonstrate durable immune tolerance or clinical independence from immunosuppression.

### 6.4 Immune engineering of transplanted cells is an emerging strategy

Cell-intrinsic immune engineering changes how a graft is recognised rather than placing a barrier around it. The design problem is multi-objective. It must reduce allogeneic T-cell recognition without exposing the cells to natural killer-cell or macrophage attack. It must also preserve endocrine identity and glucose-responsive insulin secretion.

Cell-intrinsic protection can target susceptibility to autoimmune injury as well as antigen presentation. Cai et al. used an *in vivo* CRISPR screen to identify RNLS as a beta-cell protection target in T1D models (54). Bode et al. subsequently linked Rnls deficiency to changes in NK-cell regulatory activity (55). These mechanistic findings support protection against particular immune stresses, but do not establish resistance to allogeneic rejection in humans.

The limits of single edits are illustrated by a study in which RNLS or HIVEP2 knockout did not prevent clearance of beta-cell spheroids in immunocompetent allogeneic or xenogeneic hosts (56). These experiments used murine and human beta-cell lines rather than primary clinical islet products. Their results support combining complementary protective mechanisms, while showing why an edit that protects against autoimmunity cannot be assumed to provide broad transplant immune privilege.

Early hypoimmune platforms combined deletion of beta-2-microglobulin and the class II major histocompatibility complex transactivator with CD47 expression (32). More selective deletion of polymorphic HLA molecules has also reduced immune recognition in stem-cell-derived islets while retaining endocrine differentiation (14). These studies show that immune compatibility can be engineered without necessarily sacrificing the therapeutic phenotype.

Single edits are generally insufficient to address all relevant immune pathways simultaneously. Complete loss of classical HLA class I may reduce T-cell recognition but can increase susceptibility to natural killer-cell responses. More advanced studies showed that engineered human islets resisted both allogeneic rejection and autoimmune injury in humanised mice (37). Hypoimmune allogeneic islets also achieved insulin independence without systemic immunosuppression in a small diabetic non-human-primate experiment (38). This strategy has now entered early clinical testing. In a first-in-human case, CRISPR–Cas12b-edited and lentivirally modified allogeneic donor islet cells were implanted into the forearm muscle of a man with long-standing T1D without systemic immunosuppression. At 12 weeks, the cells showed stable, glucose-responsive C-peptide secretion without a detectable immune response against the edited graft (57). This finding provides clinical proof of concept for immune-evasive allogeneic beta-cell transplantation, but the small graft dose, single participant and short follow-up preclude conclusions about insulin independence, long-term immune escape or safety. Together, these findings provide strong preclinical evidence and initial human proof of concept, but not evidence of therapeutic-dose efficacy or broad clinical durability.

Immune-evasive products require a higher safety standard, not a lower one. Genetic stability, tumour control and a practical way to remove the graft must be built into the development programme. A stem-cell platform that combined beta-cell selection with two independent safety mechanisms provides one example of this principle (27). Preimplantation testing alone cannot resolve the long-term safety of cells designed to persist while avoiding immune clearance. Multiplex designs must therefore be evaluated as complete products, with both immune resistance and glucose-responsive function tested after editing; adding edits is not itself evidence of greater safety or clinical efficacy (56, 58).

### 6.5 Regulatory T cells may support immune tolerance

Regulatory T cells (Tregs) are best considered adjuncts rather than replacement products. Their potential role is to restrain pathogenic immune responses and improve conditions for engraftment (59–61). This may be especially relevant when recurrent autoimmunity remains active despite control of allorecognition.

Clinical studies establish feasibility but not efficacy. In a phase 1 study, expanded autologous regulatory T cells were well tolerated in adults with recent-onset T1D (21). A separate small study combined autologous polyclonal regulatory T cells with intraportal allogeneic islets (62). Neither result shows that regulatory T cells can replace chronic immunosuppression or prevent recurrent autoimmune injury on their own.

Antigen-specific and chimeric antigen receptor regulatory T-cell approaches aim to direct suppression more precisely to the graft. In a preclinical islet-autoimmunity model, immune conditioning followed by antigen-specific regulatory T cells controlled injury more effectively than either component alone (63). A related platform generated CAR regulatory T cells directed to an inert ligand expressed by engineered human pluripotent stem-cell derivatives (64). These approaches remain preclinical and must be judged by durable graft function, not circulating regulatory-cell numbers alone. Endogenous Treg recruitment through local signals offers a distinct adjunctive route (Section 6.3). For both recruited and infused Tregs, graft localisation, suppressive function, persistence and metabolic benefit require assessment; cell numbers alone are insufficient evidence of tolerance (53, 59).

An integrated system may combine a defined endocrine graft, a suitable implantation niche and targeted immune protection. Each component should be tested for the pressure it addresses and the new burden it introduces. This is a mechanistic integration question: every strategy should therefore be judged by its ability to deliver biological benefit without shifting an excessive burden to the recipient. Systemic immunosuppression has the most established human evidence, whereas non-isolating materials and engineered Tregs remain predominantly preclinical. Encapsulation and cell-intrinsic editing have reached human testing, but efficacy and durability are product-specific. Combinations may address complementary barriers, provided their added surgical, manufacturing and safety burdens are measured (**Table 1**).

## 7 Translational and clinical progress

Clinical studies now support beta-cell replacement as more than a theoretical goal. They also show why reported outcomes must be interpreted in the context of the whole treatment system. Cell source, differentiation stage, implantation site, immune treatment and follow-up differ markedly between studies.

### 7.1 Donor islet transplantation has established clinical feasibility

Donor-islet transplantation has established that endogenous insulin secretion can be restored in people with T1D (22, 65). Its clearest clinical role is in selected people with recurrent severe hypoglycaemia despite optimised care. In the phase 3 Clinical Islet Transplantation Consortium study, 87.5% of participants met the composite endpoint of HbA1c below 7.0% without severe hypoglycaemic events at one year. At two years, 71% retained this outcome (6).

This evidence also shows why insulin independence is an incomplete endpoint. Detectable C-peptide indicates graft activity, while prevention of severe hypoglycaemia shows that the activity has clinical value. Insulin independence is more demanding and may be lost even when residual graft function continues to improve glucose stability.

Long-term experience makes this distinction clear. In a 20-year single-centre cohort of 255 recipients, 79% achieved insulin independence at some point. The estimated proportion remaining insulin-independent declined to 32% at five years and 20% at ten years (8). Donor-islet transplantation therefore establishes clinical feasibility, but it also defines the need for more durable products and safer immune protection.

### 7.2 Pluripotent stem-cell-derived products have entered early clinical development

Early clinical studies of pluripotent stem-cell (PSC)-derived products provide human evidence of graft survival, maturation and endocrine activity. They test different therapeutic systems, however, and should not be interpreted as interchangeable evidence. The product format, cell dose, transplantation site and immune regimen all affect what a result can show (**Table 2**).

**Table 2:** Clinical and translational evidence for donor- and stem-cell-derived β-cell replacement in type 1 diabetes.

Product, study design and evidence stage	Participants/delivery	Immune regimen	Follow-up and key efficacy finding	Interpretive boundary/safety signal
Donor islets: CIT-07, phase 3 (NCT00434811) (6)	48 adults with T1D and severe hypoglycaemia; intraportal islet infusion.	Systemic immunosuppression.	At 1 year, 87.5% met the composite endpoint of HbA1c <7.0% without severe hypoglycaemia; 71% retained it at 2 years.	Clinical proof of principle, but donor supply and immunosuppression constrain broad application. Serious events included cytopenias, infections and renal dysfunction.
Donor islets: single-centre observational 20-year cohort (8)	255 recipients with T1D; clinical islet transplantation.	Systemic immunosuppression.	79% achieved insulin independence at some point; estimated insulin independence was 32% at 5 years and 20% at 10 years.	Shows that insulin independence can decline despite initial graft function; long-term durability must be reported.
PSC pancreatic endoderm: PEC-01 in VC-02/PEC-Direct, open-label phase 1/2 (NCT03163511) (26)	15 participants at one trial site; subcutaneous vascularising, non-immunoprotective macrodevice.	Systemic immunosuppression.	One-year analysis showed *in vivo* maturation and fasting and glucose-responsive C-peptide secretion.	Demonstrates engraftment and maturation, not complete insulin replacement.
Autologous chemically induced iPSC-derived islets: first-in-human case report (31)	One participant with T1D; implanted beneath the abdominal anterior rectus sheath.	Continued background immunosuppression following prior liver transplantation; no allogeneic immune mismatch.	Insulin independence began 75 days after transplantation and persisted through 1 year.	Single case; recurrent autoimmunity remains relevant, and the report did not test immune-treatment independence, scalable manufacture, population-level durability or long-term safety.
Fully differentiated allogeneic PSC-derived islets: VX-880 (zimislecel), reported phase 1/2 cohort (NCT04786262) (5)	14 participants with ≥12 months of follow-up; portal-vein infusion.	Systemic immunosuppression.	All developed detectable C-peptide. At day 365, 12 full-dose recipients had no severe hypoglycaemia and HbA1c <7%; 10 were insulin-independent.	Multicentre early-phase evidence for a manufactured allogeneic product, but not a treatment-free cure. Neutropenia was the most common serious adverse event.
Fully differentiated PSC-derived islets plus immune-isolating device: VX-264, phase 1/2; sponsor update (49).	Parts A/B enrolled and dosed; participant count not specified in the cited announcement.	Designed to avoid systemic immunosuppression.	Day-90 Part B analysis: C-peptide increases did not meet the efficacy endpoint. Further clinical development was discontinued in March 2025.	Sponsor reported generally acceptable short-term safety/tolerability; no proven clinical benefit or long-term safety. Mechanism of inadequate function not established by this announcement.
Gene-edited allogeneic donor islet cells: first-in-human case (57).	One man with long-standing T1D; implantation into forearm muscle.	No systemic immunosuppression.	At 12 weeks, the graft showed stable, glucose-responsive C-peptide secretion without a detectable immune response against the edited cells.	Subtherapeutic graft dose and single participant; no insulin independence demonstrated; long-term efficacy, immune escape and safety remain unknown.

Evidence note: stages refer to the cited reports, not necessarily the current recruitment status. The VX-264 entry is based on a sponsor announcement dated 28 March 2025, checked on 15 September 2026. Sample size, dose, follow-up and background immunosuppression limit cross-study comparisons. C-peptide secretion, insulin independence and independence from immune treatment are separate outcomes.

One route used PEC-01 pancreatic endoderm in the subcutaneous, vascularising VC-02/PEC-Direct device with systemic immunosuppression (NCT03163511). In a one-year analysis of 15 participants at one site of an open-label phase 1/2 trial, the implanted cells developed a mature beta-cell phenotype and produced fasting and glucose-responsive C-peptide (26). This study demonstrated engraftment and *in vivo* maturation, rather than complete insulin replacement.

A second route implanted autologous chemically induced iPSC-derived islets beneath the abdominal anterior rectus sheath. The reported participant became insulin-independent 75 days after transplantation and remained so through one year (31). This result is encouraging, but one case cannot establish reproducible manufacture, population-level durability or protection from recurrent autoimmunity.

Among the clinical studies included in this review, a multicentre phase 1/2 study evaluated fully differentiated allogeneic stem-cell-derived islets delivered by portal-vein infusion. All 14 participants with at least 12 months of follow-up developed detectable C-peptide. At day 365, all 12 full-dose recipients were free from severe hypoglycaemia and had HbA1c below 7%; 10 were insulin-independent (5). The study shows substantial endocrine function from a manufactured allogeneic product. It does not establish long-term durability or a treatment-free cure because participants received systemic immunosuppression.

These studies represent distinct stages of translation rather than a simple competition between platforms. Pancreatic endoderm studies address human maturation. Autologous iPSC studies address individualised cell replacement. Allogeneic studies address the potential for a banked manufactured product. No study has yet resolved every problem at once. The evidence in **Table 2** spans long-term donor-islet cohorts, small early-phase trials and individual cases. Small, uncontrolled studies cannot establish comparative efficacy, and short follow-up is particularly limiting for immune-engineered or PSC-derived products. The negative VX-264 update also warrants inclusion, with its sponsor-reported status distinguished from peer-reviewed trial evidence (49).

### 7.3 Clinical translation requires integration of multiple elements

Clinical benefit should be assessed across five related domains: graft function, clinical benefit, durability, treatment burden and long-term safety (**Figure 3**). These domains are interdependent and should be considered together rather than as a strictly sequential pathway. Graft function alone is insufficient. Dynamic C-peptide should demonstrate physiological glucose responsiveness, while CGM measures, HbA1c, severe-hypoglycaemia events and insulin requirements indicate whether graft activity provides meaningful clinical benefit.

**Figure 3:**
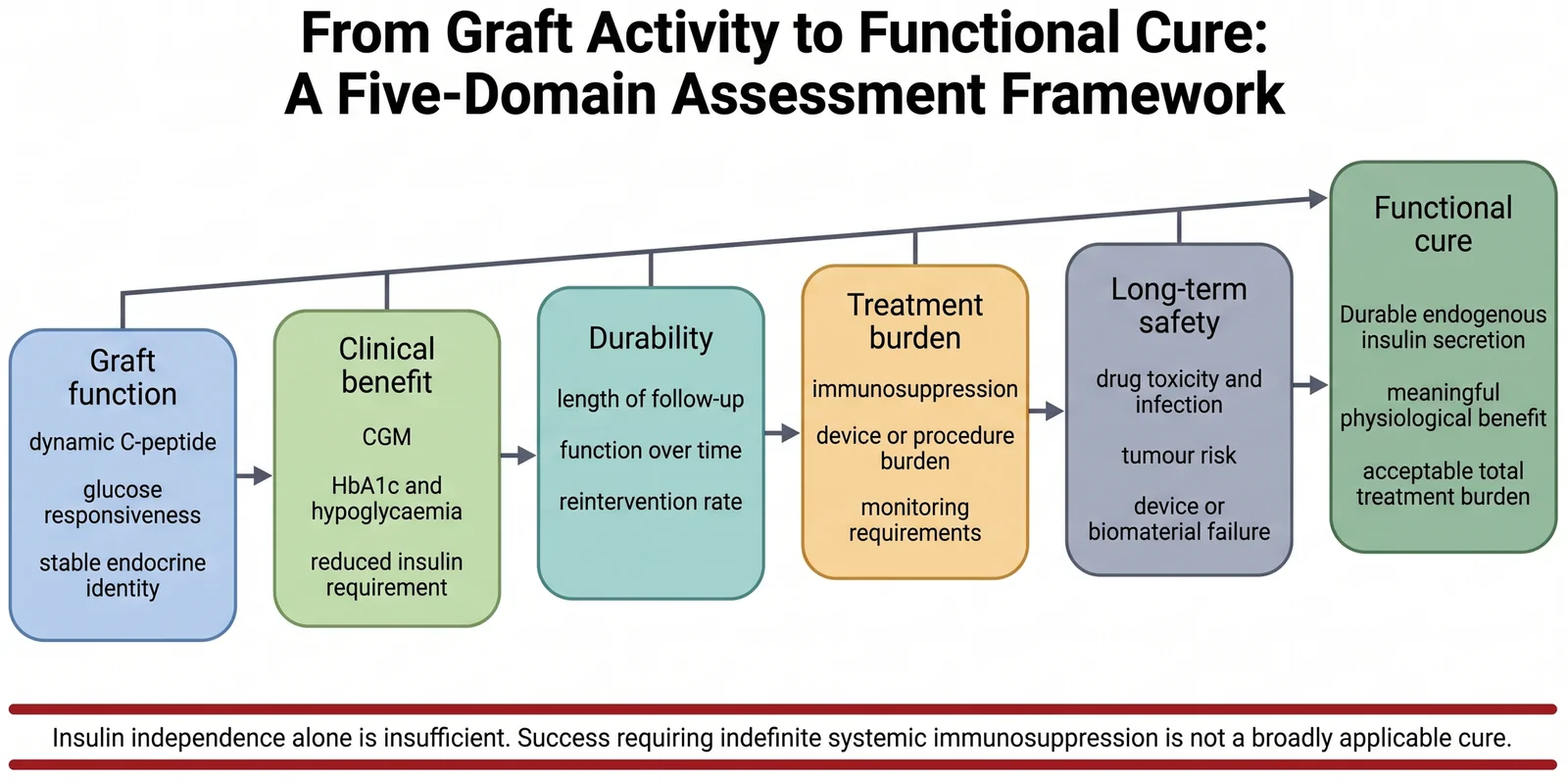
From graft activity to functional cure: a five-domain assessment framework. A favourable endpoint alone is insufficient; graft function, clinical benefit, durability, treatment burden and long-term safety must be assessed together.Tables.

Durability must be part of the definition of success rather than a later qualification. One-year insulin independence is important evidence of efficacy, but it cannot establish lasting graft function. Reports should state how benefit changes with follow-up and whether insulin is restarted after partial graft decline. They should also distinguish insulin reduction from insulin independence.

Treatment burden and long-term safety should be assessed as distinct but related domains. Systemic immunosuppression, implantation and repeat procedures, device maintenance, intensive monitoring and rescue interventions all contribute to treatment burden. Long-term safety assessment should include procedure-related complications, infection, immune toxicity, abnormal cell growth and device or biomaterial failure.

Beyond these five outcome domains, manufacturing and access determine whether an effective treatment can be translated into routine clinical practice. Autologous iPSC products may reduce classical allorecognition, but personalised manufacture must justify its time, cost and release requirements. Banked allogeneic products may be easier to scale, but they require durable control of alloreactivity. Claims of scalability should therefore include manufacturing success, product release failures, storage, transport and continuing specialist care.

Patient selection is another cross-cutting translational consideration. The benefit-risk balance differs between a person with recurrent severe hypoglycaemia and a person with stable glucose control on optimised therapy. Immune history may also influence outcome, as HLA compatibility and post-transplant alloantibody and autoantibody responses have been associated with graft survival or clinical outcome (35, 36). These observations do not provide a complete selection algorithm, but they argue against treating recipients as immunologically uniform.

In this review, functional cure is a working definition rather than a field-wide consensus. It requires durable endogenous insulin secretion that provides meaningful physiological benefit at an acceptable total treatment burden. A claim of durability must report the length and pattern of follow-up; one-year insulin independence alone does not establish it. At minimum, clinical reports should include dynamic C-peptide, CGM measures, HbA1c, severe-hypoglycaemia events and exogenous insulin requirement. They should also report reintervention, immunosuppression or device burden, and long-term safety. Together, these five domains allow cell products and immune-protection approaches to be compared as complete therapeutic systems (**Figure 3**).

## 8 Challenges and future directions

The remaining challenge is to test integrated replacement platforms in clinically relevant forms. The following priorities concern future trial design, product development, patient selection, long-term safety and equitable access.

### 8.1 Integrated replacement-protection platforms are likely to be necessary

Preclinical studies often examine the cell product, implantation niche and immune intervention separately. This is useful for mechanism, but it can overestimate clinical readiness. The final product should be tested in the form intended for use, including its dose, site, biomaterial and immune regimen.

No model reproduces every feature of human T1D. Complementary models are therefore needed, with each model assigned a clear purpose. Early studies should measure cell survival, vascularisation and innate injury. Later studies should test alloreactivity, autoimmune recurrence, metabolic function and the safety of the complete system.

### 8.2 Improving maturation and functional stability remains a priority

Insulin-positive cells are not necessarily a clinically useful endocrine product. The relevant question is whether the graft can sense glucose, process proinsulin and release insulin with stable kinetics after transplantation. Marker expression and short-term glucose responses should be interpreted as supporting evidence, not as proof of durable function.

Product maturation should be evaluated together with stress tolerance. Hypoxia, inflammation and limited nutrient supply can expose functional weaknesses that are not visible in standard culture. Comparisons should therefore include the post-transplant environment that the product is expected to face.

### 8.3 Precision selection of products and patients may improve outcomes

The acceptable benefit-risk balance is not identical for all people with T1D. A treatment requiring systemic immunosuppression may be justified for a person with recurrent severe hypoglycaemia despite optimised care. The same burden may not be justified for someone who can manage glucose safely with current treatment.

Trials should report the clinical features that define the intended population. Relevant factors include severe-hypoglycaemia history, residual endogenous function, comorbidities, prior transplantation and immune risk. Product selection should also reflect the desired speed of insulin delivery, need for graft retrievability and acceptable level of immune treatment.

### 8.4 Manufacturing and regulatory scalability will determine clinical impact

A product cannot have broad clinical impact unless it can be manufactured consistently. Release testing must address potency, cell composition, residual undifferentiated cells, genetic stability, storage and transport. For immune-engineered products, it must also assess recognition by T cells, natural killer cells and macrophages (14, 27, 32, 37, 38).

Regulatory evaluation should follow the product through its full life cycle. This includes cell-line provenance, batch manufacture, implantation, delayed adverse events and a feasible way to monitor or remove the graft. Cost, specialist infrastructure and geographic access will also determine whether a successful platform remains limited to a few centres or becomes a realistic treatment option.

### 8.5 Long-term safety requires product-specific risk mitigation

Residual undifferentiated PSCs and unwanted proliferative populations create risks of teratoma formation or ectopic tissue growth. These should be distinguished from malignant transformation associated with acquired genetic changes. Product purification, sensitive assays for residual unwanted cells and preclinical tumourigenicity testing are complementary safeguards; a negative assay must be interpreted against its detection limit and the intended cell dose (27, 66).

Genome editing introduces additional concerns, including unintended on-target changes, off-target alterations and chromosomal rearrangements. Integrating vectors require consideration of insertion-related effects. Genomic characterisation should accompany testing of cell identity, growth and endocrine potency, because an apparently protective edit may alter beta-cell function. Multiplex engineering increases the need to examine the final clone and manufactured product rather than validating each edit in isolation (56, 58).

Immune-evasive cells also raise the possibility of reduced clearance if abnormal growth develops. Preclinical safety-switch strategies, including the double fail-safe platform described above, offer a potential route to eliminate unwanted cells, but require validation in the intended product (27). Accessible implantation sites or retrievable devices may facilitate intervention, although complete graft removal cannot be assumed. Long-term monitoring should address abnormal growth, altered hormone secretion, graft failure and procedure- or device-related complications. Surveillance and rescue plans should be tailored to the product and route; brief tumour-free follow-up in a small cohort cannot establish long-term safety (46, 67).

### 8.6 Ethical considerations and equitable access

Consent for early-phase trials must explain uncertain benefit, delayed risks and prolonged follow-up. For gene-edited grafts, withdrawing from a study does not necessarily remove the implanted cells or reverse their genetic changes. Discussions should address practical limits to retrieval and available alternatives. hESC-derived products additionally require transparent cell-line provenance and attention to embryo-related values and donor consent (66).

High manufacturing costs, specialist delivery and continuing monitoring may restrict access even if cell supply becomes scalable. Development should therefore consider affordability, geographic access and transparent, clinically justified eligibility criteria alongside efficacy. These considerations are particularly important when discussing a potential functional cure, which should not imply either assured benefit or broad availability before these have been demonstrated (66).

Progress will be most convincing when these questions are addressed together. A mature graft, effective immune protection and scalable manufacture are interdependent requirements. The next generation of trials should make their trade-offs explicit rather than presenting a favourable endpoint in isolation.

## 9 Conclusion

Beta-cell replacement has moved from proof of concept to early clinical reality. Donor-islet transplantation shows that restored endogenous insulin secretion can reduce severe hypoglycaemia in selected people with T1D. Stem-cell-derived products have also shown human graft function, glucose-responsive C-peptide secretion and, in some recipients, insulin independence (5, 6).

These findings establish clinical potential, not a broadly applicable cure. Current success remains limited by early graft loss, incomplete product maturity, alloimmune and autoimmune injury, and the burden of maintaining graft function. The most encouraging studies should therefore be interpreted as evidence for a therapeutic platform that is still being defined.

A functional cure calls for stable endogenous insulin secretion that provides real physiological benefit without imposing an excessive long-term burden. Short-term insulin independence alone is not enough. Neither is a result sustained only by indefinite systemic immunosuppression. Progress will depend on integrating a reliable cell product, a supportive implantation niche and targeted immune protection into a treatment that can be manufactured, monitored and safely used at scale.
