# Supplementary material for: Beta-cell replacement for type 1 diabetes: integrating cell products, engraftment and immune protection
Source: Front Endocrinol (Lausanne). 2026 Oct 5;17:1981945. doi: 10.3389/fendo.2026.1981945 (PMC13678449; doi:10.3389/fendo.2026.1981945)
Supplement: Supplementary file 1 [file SupplementaryFile1.docx]

Pubmed: 1531

(((Diabetes Mellitus, Type 1 ) OR (Autoimmune Diabetes) OR (Diabetes Mellitus, Brittle) OR (Diabetes Mellitus, Insulin-Dependent) OR (Diabetes Mellitus, Insulin-Dependent, 1) OR (Diabetes Mellitus, Juvenile-Onset) OR (Diabetes Mellitus, Ketosis-Prone) OR (Diabetes Mellitus, Sudden-Onset) OR (Diabetes Mellitus, Type I) OR (Diabetes, Autoimmune) OR (IDDM) OR (Insulin-Dependent Diabetes Mellitus 1) OR (Juvenile-Onset Diabetes) OR (Type 1 Diabetes) OR (Type 1 Diabetes Mellitus)) AND ((Insulin-Secreting Cells) OR (Pancreatic B Cells) OR (Pancreatic beta Cells) OR (beta Cells, Pancreatic) OR (Islets of Langerhans) OR (Islands of Langerhans) OR (Islet Cells) OR (Nesidioblasts) OR (Pancreas, Endocrine) OR (Pancreatic Islets))) AND ((Cell Transplantation) OR (Islets of Langerhans Transplantation) OR (Stem Cell Transplantation) OR (Pluripotent Stem Cells) OR (Embryonic Stem Cells) OR (Human Embryonic Stem Cells) OR (Induced Pluripotent Stem Cells) OR (Graft Survival) OR (Neovascularization, Physiologic) OR (Immunomodulation) OR (Immunosuppression Therapy) OR (Transplantation Tolerance) OR (Graft Rejection) OR (Cell Encapsulation) OR (Biocompatible Materials) OR (Cell Engineering) OR (Genetic Engineering) OR (Gene Editing) OR (Lymphocytes, Regulatory) OR (beta-cell replacement) OR (stem-cell-derived islet) OR (stem-cell-derived beta cell) OR (engraftment) OR (implantation) OR (immune protection) OR (hypoimmune) OR (hypoimmunogenic) OR (immune-evasive) OR (CAR-Treg)) Filters: Adaptive Clinical Trial, Books and Documents, Case Reports, Clinical Study, Clinical Trial, Clinical Trial, Phase I, Clinical Trial, Phase II, Clinical Trial, Phase III, Clinical Trial, Phase IV, Comparative Study, Conference Proceedings, Consensus Statement, Controlled Clinical Trial, Corrected and Republished Article, Dataset, Duplicate Publication, Electronic Supplementary Materials, Evaluation Study, Practice Guideline, Pragmatic Clinical Trial, Preprint, Published Erratum, Randomized Controlled Trial, Randomized Controlled Trial, Veterinary, Twin Study, Validation Study, Clinical Trial, Veterinary

WOS: 872

#1: "Diabetes Mellitus, Type 1 " OR "Autoimmune Diabetes" OR "Diabetes Mellitus, Brittle" OR "Diabetes Mellitus, Insulin-Dependent" OR "Diabetes Mellitus, Insulin-Dependent, 1" OR "Diabetes Mellitus, Juvenile-Onset" OR "Diabetes Mellitus, Ketosis-Prone" OR "Diabetes Mellitus, Sudden-Onset" OR "Diabetes Mellitus, Type I" OR "Diabetes, Autoimmune" OR "IDDM" OR "Insulin-Dependent Diabetes Mellitus 1" OR "Juvenile-Onset Diabetes" OR "Type 1 Diabetes" OR "Type 1 Diabetes Mellitus"

# #2: "Insulin-Secreting Cells" OR "Pancreatic B Cells" OR "Pancreatic beta Cells" OR "beta Cells, Pancreatic" OR "Islets of Langerhans" OR "Islands of Langerhans" OR "Islet Cells" OR "Nesidioblasts" OR "Pancreas, Endocrine" OR "Pancreatic Islets"

#3: "Cell Transplantation" OR "Islets of Langerhans Transplantation" OR "Stem Cell Transplantation" OR "Pluripotent Stem Cells" OR "Embryonic Stem Cells" OR "Human Embryonic Stem Cells" OR "Induced Pluripotent Stem Cells" OR "Graft Survival" OR "Neovascularization, Physiologic" OR "Immunomodulation" OR "Immunosuppression Therapy" OR "Transplantation Tolerance" OR "Graft Rejection" OR "Cell Encapsulation" OR "Biocompatible Materials" OR "Cell Engineering" OR "Genetic Engineering" OR "Gene Editing" OR "Lymphocytes, Regulatory" OR "beta-cell replacement" OR "stem-cell-derived islet" OR "stem-cell-derived beta cell" OR "engraftment" OR "implantation" OR "immune protection" OR "hypoimmune" OR "hypoimmunogenic" OR "immune-evasive" OR "CAR-Treg"

#3 AND #2 AND #1

Emabse: 993

1	insulin dependent diabetes mellitus/	179031

2	pancreas islet beta cell/	56353

3	(Cell Transplantation or Islets of Langerhans Transplantation or Stem Cell Transplantation or Pluripotent Stem Cells or Embryonic Stem Cells or Human Embryonic Stem Cells or Induced Pluripotent Stem Cells or Graft Survival or Neovascularization, Physiologic or Immunomodulation or Immunosuppression Therapy or Transplantation Tolerance or Graft Rejection or Cell Encapsulation or Biocompatible Materials or Cell Engineering or Genetic Engineering or Gene Editing or Lymphocytes, Regulatory or beta-cell replacement or stem-cell-derived islet or stem-cell-derived beta cell or engraftment or implantation or immune protection or hypoimmune or hypoimmunogenic or immune-evasive or CAR-Treg).ab,ti,kw.	749878

4	1 and 2 and 3	993
